# Can selfies trigger social anxiety? A study on the relationship between social media selfie behavior and social anxiety in Chinese youth group

**DOI:** 10.3389/fpsyg.2022.1016538

**Published:** 2022-11-15

**Authors:** Yixuan Liu, Jiayu Zhu, Jianping He

**Affiliations:** School of Media and Communication, Center for Media and Cultural Development, Shenzhen University, Shenzhen, China

**Keywords:** selfie behavior, social anxiety, social comparison, body image, youth groups

## Abstract

As modernization continues to advance the development of digital society, social media has become an important part of people’s daily life and an extension and expansion of real social interactions. In this process, social media use and individual social psychology have increasingly become the object of academic attention, among which the relationship between selfie behavior, as an important interaction practice of youth group in social media, and social anxiety needs to be further explored and discussed. The purpose of this study is to investigate the current situation of selfie behavior, body image, and social anxiety among young people in China. Using a combination of qualitative and quantitative empirical methods, a questionnaire survey was conducted in Chinese mainland (*n* = 920) to examine the mediating effects of social comparison and body image on social media selfie behavior and social anxiety, and found that there was a significant negative relationship between youth social media selfie behavior and social anxiety, while the sequence mediating effects of social comparison and body image were significant. The findings of the study provide new ideas and directions for exploring the intervention paths of youth social psychology in the era of image socialization.

## Introduction

Along with the development of social media, social media has integrated a variety of information dissemination methods, changed the form of human interaction in interpersonal communication, and constructed a real online virtual space. Through the use of social media, more and more people have been given the opportunity to participate in culture, consume and produce, and in these opportunities, they have realized the construction of their self-image. For the youth group, their self-presentation in social media is accompanied by purpose and selectivity (Pang, 2020), “Selfie” is an important means of self-presentation and impression management for the youth group (Lee and Lee, 2019). In the digital space, the digitized body is an important way of presenting individual body image, for example, individuals record their appearance, physique and behavior through selfie photos or videos, which is a common performance of digitized body in digital existence. The selfie image has become a new expression of their life and learning, social sharing, and leisure, reflecting the release of the desire for self-expression of the youth group in the social media environment, and a new social strategy of digital body participation in social interaction (Tiggemann et al., 2020). The so-called selfie refers to individuals taking pictures of themselves through devices with camera devices such as digital cameras, smartphones, and tablets, and the act of posting refers to individuals posting their selfie photos on social media for display, recording, or sharing, and receiving likes and comments, thus forming a selfie cycle. Some individuals also choose to make elaborate adjustments and retouch their selfies through digital editing technology in order to achieve a better presentation. Today, selfies have become a common and popular cross-cultural trend (Shir and Yair, 2017).

However, with the immersion, popularity and anxiety brought about by the phenomenon of selfies, the social problems resulting from the image of an individual’s appearance and related body perceptions have proliferated, not only affecting the formation of immature esthetics and aberrant psychology in youth group, but also leading to reduced well-being among family parents and children, negative body satisfaction and negative social psychology, among others (Verduyn et al., 2015), which has sparked widespread concern in the community. According to the social comparison theory proposed by Festinger, the level of social media participation will cause individuals to have a high level of social comparison psychology, especially the self-representation behavior in social media will make individuals pay more attention to their appearance and others’ evaluation of their body condition, and individuals with a high level of social comparison psychology will affect the formation of negative body image, and thus individuals’ concerns about the negative evaluation given by others during the social process. This can lead to social anxiety due to concerns and fears about the negative evaluations given by others during social interaction (Festinger, 1954). In particular, the current global pandemic of Corona Virus Disease (COVID-19) that emerged in early 2020 is not over, with occasional outbreaks of various scales around the world, and the highly contagious nature of the virus has had a significant impact on people’s daily social lives. The Chinese government implemented strict universal travel restrictions in early 2020, including measures such as home quarantine, stopping offline teaching, and implementing online teaching, which severely affected the psychological and emotional well-being of the youth population (Wang and Zhao, 2020). In this social context, where social activities are gradually shifting to online media and people’s social connections are shifting from offline to online, it is common for people to feel nervous, anxious, or upset in the face of sudden lifestyle changes (Chen et al., 2020). Therefore, this study aims to investigate the social anxiety of a specific group of individuals online, to investigate the relationship between online specific self-representation behaviors and social anxiety, and to verify the serial mediation effects of social comparison and body image. By examining youth selfie behaviors and social anxiety, the relationship between individual social media selfie behaviors, body image, and social anxiety was clarified. The investigation of this study helps to theoretically clarify the underlying psychological mechanisms of how selfie behaviors affect social anxiety in youth group, and provides a theoretical basis for the prevention and intervention of adverse social emotions such as negative body perceptions in youth. The detailed discussion of the relationship between selfie behavior and social anxiety is an important issue of information communication effects in the post-industrial era, and this proposition is not only related to the development path of human mediated society, but also has its unique theoretical significance and practical value.

## Literature review

### Technologically mediated social anxiety: Research findings and controversies

Anxiety is a negative emotional state that arises when people anticipate something bad or threatening, often accompanied by nervousness and fear. The concept of social anxiety was originally thought to consist of evaluation anxiety and avoidance and distress, representing the individual’s fear of negative evaluation by others and the individual’s avoidance of social behavior in real social situations, respectively, (Watson and Friend, 1969). The emergence of social media has expanded people’s social interactions and provided new opportunities for impression management and mutual communication. People can selectively express themselves by choosing photos and emoticons on social media, making self-reinvention possible. For impression management purposes, social media enables individuals to regularly present themselves in a variety of ways that enhance their sense of self-esteem (Pounders et al., 2016), is beneficial in helping individuals shape their self-identity and present a positive and desirable side of themselves (Verduyn et al., 2020), as well as providing fields for self-disclosure, positive interactions with peers and spread social connections (Verduyn et al., 2015), helping individuals to gain positive online social support (Antoci et al., 2015).

However, the more empirical findings of social anxiety research under social media are not promising. First of all, previous studies have demonstrated that social media is beneficial in enhancing individuals’ self-efficacy in impression management, but it does not mean that social media use reduces social anxiety. A study that empirically investigated adolescents and young adults showed that social media engagement and behaviors, especially activities involving appearance comparisons and judgments, are more likely to lead to depression and social anxiety symptoms (Hawes et al., 2020). Although social media can alleviate realistic stress, it can become a tool for people to escape from reality, impair the development of individual social skills, and can cause individuals to experience stronger anxiety in realistic interactions. Secondly, many individuals with high levels of social anxiety in reality are still wired with social anxiety characteristics during social media use, and online activities can make social anxiety more pronounced (Weidman and Levinson, 2015). Also browsing information in social media can make individuals overly concerned with the behavioral performance of the self in social situations, the more severe the tendency of social anxiety (Vannucci et al., 2017). Not only social media, but also time spent on games and social anxiety are positively correlated (Prizant-Passal et al., 2016). It has also been proven that frequent users of social media develop irrational beliefs such as others are better and happier than they are, and can have psychological emergence such as social comparison (De Vries and Kühne, 2015), and this leads to emotional experiences such as anxiety and depression (Shaw et al., 2015).

### Current status of research on selfie behavior

According to the impression management theory proposed by Goffman, people want to control their image and identity-related information in social settings (Rodgers, 2016), and posting selfies is an efficient way to express themselves and manage their impressions (Bayer et al., 2020). Research exploring the motivations for posting selfies also suggests that people care about the opinions of others in social networks and will construct and manage impressions by presenting their positive selves (Sung et al., 2016). Impression management theory also suggests that managing an image often involves both “onstage presentation” and “behind-the-scenes preparation” (Goffman, 1959), and this is also true for selfies. Therefore, posting selfies on social media is not a single act, but a continuous process consisting of multiple acts. Specifically, in addition to the “pre-posting” act of posting selfies, there are also “behind-the-scenes” preparatory acts such as selecting and editing selfies before posting them (De Vaate et al., 2018). Three types of activities are commonly associated with selfies: taking selfies, posting selfies, and editing selfies (Dhir et al., 2016). It has also been noted that photo investment and photo manipulation are also two aspects of photo posting (Mclean et al., 2015). Among them, photo investment reflects one’s concern about the quality of the photo and the effort in selecting self-photographs prior to relevant sharing, such as the time to select the photo. Photo manipulation, which is related to photo investment, refers to changing photo elements before sharing, such as the person’s features, e.g., fixing oneself to be thinner. Previous research has found that information posting and information viewing behaviors on social media can have different psychological effects. Researchers have found similar phenomena in the context of selfies, for example, viewing selfies and posting selfies were negatively associated with body self-esteem (Chang et al., 2019).

The current academic research on selfies focuses on two aspects: on the one hand, the relationship between self-personality characteristics and selfie behavior, and on the other hand, people’s perceptions and opinions about selfies. The existing studies mainly focus on the frequency of posting selfie photos on social media, with few relevant questions and single content, ignoring the degree of input of selfie individuals to the photos, and the current research on selfie behavior lacks precise and effective measurement tools. Foreign studies have also begun to focus on the adverse effects of selfie behavior on individuals, such as selfie addiction caused by excessive selfies (Starcevic et al., 2018), and the effects of female selfie behavior on self-objectifcation and mental health among others (Cohen et al., 2018). However, there is still a lack of systematic empirical research on selfie behavior and less involvement in the adverse effects of selfies on individuals. Therefore, the present study explores the mechanisms of influence related to selfie behavior and social anxiety, filling a gap in related research.

### Related studies on social comparison

The social comparison theory was first put forward in 1954. It is believed that everyone has the viewpoint and ability to evaluate himself. This evaluation needs to be carried out under the condition that the outside world has certain reference systems and objective standards. If there is no such evaluation standard in the existing environment, individuals tend to compare with others in order to enhance the accuracy of their own evaluation (Festinger et al., 1954). Individuals pay more attention to the results of comparison between themselves and the outside world. Generally, after social comparison, if individuals are better than others and are better than others in terms of resources, wealth, appearance and conditions, they will feel satisfied and have a sense of well-being. On the contrary, their sense of well-being will be reduced, resulting in anxiety and depression (Smith et al., 1989). With the rise of social media, this kind of social comparison has also been transferred and extended to online. Social media provides a fertile soil for people to carry out online social comparison. Its powerful function of encouraging users to actively publish personal information and widely share it with others makes the object of social comparison available through an unprecedented mass scale, which intensifies the possibility of social comparison. The research shows that the social comparison intention generated by individuals online is stronger than that offline, partly because the social media platform can help people easily express their idealized image (Verduyn et al., 2020), which provides sufficient “preparation time” for individuals. Users can strategically take selfies or other photos that can show a good life experience, and then make them public after careful editing and careful selection. However, when people browse the dynamics carefully displayed by others, they will automatically generate the idea of comparison with others, and then induce a series of negative emotions such as jealousy, loneliness and social anxiety, which seriously hinder the normal development of individual psychology (Rozgonjuk et al., 2019).

### Related studies on body image

The concept of body image was first introduced in the middle of the last century and was initially considered to be the picture that individuals construct in their minds for their own forms (Schilder, 1936). Body Image refers to an individual’s evaluation and perception of the self as a body, covering the physical features of the body and the individual’s attitude toward these features. Body image can be divided into positive and negative body image, and negative body image develops when individuals hold negative evaluations of their bodies and experience high levels of dissatisfaction. A study conducted an exploratory validation of the Multidimensional Body Image Relationship Questionnaire (MBSRQ) and concluded that body image is a multidimensional concept in which an individual’s perception and evaluation of appearance, control of thinking, emotional changes, and behavioral expressions are all manifestations of his or her body image (Cash and Fleming, 2002). The concept of “body image” is a multi-dimensional concept. Currently, scholars prefer the “multidimensional concept,” which means that an individual’s body image is formed gradually by physical, psychological, and social interactions, and the most recognized dimensions are cognition, emotion, and behavior. Generally speaking, physical appearance, body shape, functional health, emotions and feelings, and the resulting behaviors constitute body image. The complexity of the dimensions and structure of body image has given rise to many research terms related to different research directions, such as body satisfaction, positive body image, negative body image, body image disorder, etc.

## Research questions and hypothesis

### Selfie behavior and social anxiety

There is a lack of research on the impact of self-presentation behavior on social anxiety, but selfie behavior is closely related to social psychology. Some studies suggest that selfies are a way of building interpersonal relationships and gaining peer approval, and that the youth subculture is characterized by an emphasis on leisure, the importance of peers, and creativity, and that sharing selfies with peers and studying selfies is a way of building interpersonal relationships and gaining peer approval. According to the cognitive-behavioral model of social anxiety, the root of social anxiety is an individual’s assessment of the possibility of negative evaluations in social situations, i.e., when an individual enters a social situation where negative evaluations may exist, social anxiety is experienced. Therefore, when there is a discrepancy between an individual’s selfie picture and their real self, or an inflation of the mentality of social comparison, resulting in online self-presentation is associated with social anxiety (Verduyn et al., 2015). Based on this, this paper proposes the following research questions and research hypotheses.

*Research question one (R1):* What is the relationship between social media selfie behaviors and social anxiety psychology among youth group?

*Hypothesis one (H1):* Youth social media selfie behavior is positively associated with social comparison.

*Hypothesis two (H2):* Youth social media selfie behavior is positively associated with social anxiety.

### Mediating effects of social comparison

Individuals with a high degree of selfie behavior are accompanied by a high degree of photo editing behavior for a more desirable online self-presentation and to avoid receiving poor evaluations (Bell, 2019), and they are eager to seek more exposures and self-representation opportunities, and selfie photos are the key to individual impression management and self-image shaping, and they are usually more eager to obtain positive evaluations from others, and feedback from others is the key to influence self-evaluation (Wang et al., 2020). At the same time, selfies as a special self-representation behavior on social media will increase individuals’ attention to themselves, and individuals who are overly concerned with information about their physical appearance will have the psychological tendency to compare themselves with others, which will reduce their body satisfaction and generate depression (Lee and Lee, 2019). In contrast, social comparison is a process in which individuals compare themselves with others based on two dimensions of opinion and ability in order to obtain objective and accurate self-evaluation (Festinger, 1954) and social comparison in real life has a great influence on individuals’ body image and psychological mood (Gilbert et al., 1995). Based on this, the following research questions and research hypotheses are proposed in this paper.

*Research question two (R2):* Does social media selfie behavior in youth group predict higher social comparison psychology, which in turn leads to individual negative body image and thus predicts social anxiety psychology?

*Hypothesis three (H3):* Youth social comparison is positively associated with social anxiety. The higher the degree of social comparison the higher the degree of social anxiety.

*Hypothesis four (H4):* The degree of social comparison plays a mediating role in the effect of social media selfie behaviors on social anxiety in the youth group.

### The mediating effect of body image

Research in recent years has indicated that there is no association between total time spent on social media and greater levels of body image or dissatisfaction with looks, rather, it is behaviors in social media that involve appearance (e.g., selfies) that have an effect on individual body image or facial dissatisfaction (Cohen et al., 2017). Some studies have also argued for the conclusion that the more selfie activity, the more negative body image (Mclean et al., 2015). Also, related research has verified that individual body image is closely related to social anxiety. For example, individuals who are dissatisfied with the reality of their bodies produce more social avoidance behaviors (Maphis et al., 2013), and individual body monitoring predicts social anxiety (Teng and Poon, 2020). Negative body image is not a single one-sided construct, including body image dysregulation, negative body satisfaction, or distorted body image (Tylka and Wood-Barcalow, 2015), A recent study based on an empirical survey of young women concluded that negative image is positively associated with social anxiety (Bijsterbosch et al., 2020), but whether this conclusion can be extrapolated to the general youth population and whether it holds true in the Chinese context is a question that this study would like to explore further. Based on this, the following research hypothesis are proposed in this paper.

*Hypothesis five (H5):* Youth body image is negatively correlated with social anxiety. The more negative body image of youth group, the higher the level of social anxiety.

*Hypothesis six (H6):* Body image mediates the effect of social media selfie behavior on social anxiety in youth group.

### Sequential mediation effects of social comparison and body image

At the same time, according to media body image theory, the content presented by the media is often idealized and biased (Manago et al., 2008). Similarly, people tend to choose idealized content in the process of self-disclosure on social media. Similarly, people tend to choose idealized parts and embellish their images to some extent in the process of self-disclosure on social media. Second, according to social comparison theory, people tend to choose individuals with similar conditions to themselves for comparison, such as the same social class, similar family and educational background. Studies have also shown that social comparison is significantly associated with negative body image, with more social comparison leading to more body dissatisfaction and affecting the formation of positive body image, and that there are significant gender differences, with women being more likely to be influenced by social comparison than men (Myers and Crowther, 2009). Combining these two theories, individuals unconsciously generate social comparisons when engaging in behavioral activities related to selfies on social media platforms, which in turn generate dissatisfaction with their appearance, which in turn triggers negative emotions such as social anxiety (Holland and Tiggemann, 2016). Based on this, this paper proposes the following research hypothesis.

*Hypothesis seven (H7):* The degree of social comparison and body image play a sequence mediating role in the effect of social media selfie behaviors on social anxiety in youth group.

## Research method and data collection

### Research design

The study was based on the guidelines of the institutional ethics committee and met the ethical standards for relevant research. Prior to starting this study, participants were provided with information explaining the purpose of the study and after providing informed consent, they were allowed to proceed. This study used qualitative correlation method and questionnaire survey method. Firstly, in the qualitative analysis part, this study conducted participant observation and in-depth interviews on the social media selfie behaviors of the youth group to grasp their behavioral motivation and social psychology through the observation of their selfie behaviors and social behaviors. However, this observation could only provide a general understanding of their daily social behaviors and could not provide a deeper understanding of their psychological motivations. Therefore, in this paper, 20 participants with a high willingness to cooperate were selected for semi-structured in-depth interviews with research questions designed based on previous studies, and interviews were conducted both online and offline. The purpose of the qualitative study was to understand the deeper psychological motivations behind the youth group’s selfies, body image perceptions, and related social psychology. The interviews also included their experiences of social media use, social comparison and deeper psychological mechanisms of social anxiety, combining empirical and theoretical material from superficial to deep.

Before the formal interviews, the research solidarity team conducted close contact and observation with some of the respondents, and focused on observing the respondents’ selfie picture posting status on social media platforms, their online based social relationships, and interactive comments on selfie postings, in addition to relevant discussions on their daily selfie behavior. A preliminary semi-structured interview outline was developed based on the existing literature and participant observations, and a pre-study was conducted. Based on the results of the pre-study, the relevant questions were adjusted and modified, and the content to be explored in the interview outline was finally determined: the motivation of individual selfie behavior; the psychological mechanism of participating in selfie behavior; and the impact of selfie on individual social psychology.

The formal interviews lasted for 6 months, and each participant’s interview time was over 45 min. Due to the impact of epidemic prevention and control, most of the interviews were conducted through online voice calls or phone calls, and a small number of interviewees were interviewed offline by the author face-to-face. The interviews were recorded throughout and key questions were recorded based on the interviewees’ answers. During the nearly 2 months of interviews, the author also regularly observed the posting of selfie pictures on the social media platforms of the 20 interviewees in order to get a more comprehensive understanding of the interviewees’ relevant information. After completing the interviews with the 20 youths and the participatory observation on social media platforms, the researcher archived the interview materials for each interviewee in turn according to the interview time sequence, which included detailed transcripts of the interviews and the behavioral characteristics of sharing beauty photos on social media platforms. In the process of converting the audio recordings to text, the researcher carefully compared the original audio of the interviewees to ensure the accuracy of the research materials. Relevant coding and thematic analysis yielded findings in three areas of the study: selfie images as everyday social interaction, image beauty based on social comparison, and selfie-induced social anxiety.

Secondly, in the questionnaire part, the research subjects are young social media users aged 18–35 years old, because there is no unified standard for the age definition of youth group, according to WHO’s age definition of youth, people aged 18–44 years old are considered as youth group, while referring to China’s national conditions and previous related studies on youth group, the research subjects selected in this study are youth group aged 18–35 years old. The subjects selected for this study were consistent with the age range of the youth group. The study was distributed online through microblogs, WeChat, friend circles, and QQ communities, and the survey covered the entire Chinese mainland. Respondents were informed of the anonymity of the study in advance, and to ensure the semantic and structural rigor of the scale items and the convergent and discriminant validity of the items to be measured, 16 master’s and doctoral students were invited to participate in the pre-survey process, and to discuss and adjust the questionnaire for any ambiguities and misunderstandings that might arise.

### Measurement Tools

#### Selfie behavior measurement scale

The Selfie Behavior Measurement Scale was based on the frequency of individual selfies and the degree of posting by referring to the scale of posting behavior of physical appearance information in SNS compiled by scholar Lee (Lee and Lee, 2017), which measures the extent of participants’ selfie posting behavior on social media, and also adapts the scale to the actual situation of Chinese youth’s selfie posting behavior, compiling seven questions to measure individuals’ selfie posting behavior, including the specific frequency of individuals’ selfie posting behavior (e.g., the average number of selfies taken per day in the previous week, time spent on selfies), the extent of posting (e.g., posting a selfie photo, posting a visible photo at the waist and above, following others’ comments on one’s photo), the degree of integration of selfie posting behavior into one’s life, and the degree of reliance on one’s selfie. Each item was scored on a seven-point scale, and the mean value of the items was processed, with higher scores indicating higher levels of selfie posting behavior on social media. The internal consistency coefficient of this scale was also analyzed, and the *α* was 0.884, the results of exploratory factor analysis were good.

#### Social comparison measurement scale

In the present study, the questionnaire method is the most common way to measure social comparison, and the most frequently used questionnaire in the existing literature is the Social Comparison Tendency Scale proposed by Gibbons in 1999 (Gibbons and Buunk, 1999). At the same time, according to the research needs of this study, the upward appearance comparison dimension was added to the social comparison, and the Upward Appearance Comparison Scale developed by O’Brien et al. (2009), and supplemented and enriched it with relevant questions, (e.g., I compare myself to people who are better looking than me, not to people who are not as good looking as me). Each question was scored on a seven-point scale, and the mean value of the questions was processed, with higher scores indicating a higher degree of social comparison. The internal consistency coefficient analysis was also conducted for this scale, and the alpha was 0.823, with good results of exploratory factor analysis.

#### Body image measurement scale

The Multidimensional Body-Self Relations Questionnaire (MBSRQ), developed by the American psychologist Cash, is widely used in body image research, especially among young people (Flegal et al., 2007). This questionnaire is divided into appearance assessment and tendency (e.g., whether one is attractive in appearance, satisfied with one’s looks), comfort assessment and tendency (e.g., one’s feelings about physical and mental health, self-competitiveness, and stress assessment), health and illness assessment (e.g., self-judgment of one’s health and the presence of illness), body satisfaction (e.g., satisfaction with body parts and appearance assessment), overweight assessment and self-classification (e.g., perceptions of one’s own weight and determination of one’s body size as underweight or overweight). Each question item was scored according to a seven-level scale, and the mean value of the items was processed, with higher scores indicating a more positive level of body image. The internal consistency coefficients of this scale were also analyzed, and the MBSRQ had good reliability and validity for all dimensions. *α* was 0.781, with good results of exploratory factor analysis.

#### Social anxiety measurement scale

In this study, the social anxiety scale was first selected based on the Interaction Anxiety Scale (IAS) developed by Leary (1983), which measures the tendency to experience social anxiety subjectively and has been empirically tested to have good reliability and validity. In addition, Watson and Friend (1969) developed the Social Avoidance and Distress Scale (SAD; Watson and Friend, 1969), which contains two factors: social avoidance (the tendency to avoid social activities) and social distress (how individuals feel during social interactions). For the youth group, La Greca et al. (1988) designed the Social Anxiety Scale for Children (SASC; La Greca et al., 1988), which includes fear of negative evaluation, social avoidance, and distress dimensions (e.g., avoidance of social interaction; social interaction fatigue and avoidance; distress in social interaction, etc.). Each item was scored on a seven-point scale, and the mean value of the items was processed, with higher scores indicating higher levels of social anxiety. The internal consistency coefficients of this scale were also analyzed, and all dimensions of the SASC had good reliability and validity. α was 0.902, and the results of the exploratory factor analysis were good.

### Data collection

A combination of convenience sampling and snowball sampling was used in this study to conduct semi-structured in-depth interviews with 20 youths. Respondents’ occupations included teachers, students, doctors, farmers, etc. Respondents’ age range was concentrated between 19 and 32 years old, with an average age of 26 years old, and they possessed good comprehension and expression skills and enjoyed taking and uploading selfies through social media. The age range of the respondents is consistent with the youth age standard, and the significant differences in individual backgrounds within the sample are more conducive to exploring the commonalities and patterns of selfie behavior and social anxiety. To protect the privacy of the respondents, the names of the respondents appearing in the text are replaced by initials. Respondents’ specific information is presented in Table 1.

**Table 1:** Table of interviewers.

Number	Nickname	Gender	Age	Occupation
1	SXR	Male	27	Driver
2	WJL	Female	20	Student
3	WYW	Female	32	Dentist
4	LKT	Female	25	Dancer
5	ZJY	Female	31	Surgeon
6	FJS	Male	28	Journalist
7	SMH	Female	24	Nurse
8	WDN	Female	30	Attorney
9	FLW	Male	29	farmer
10	LGY	Female	22	Student
11	WYH	Male	19	Student
12	ZYZ	Female	26	farmer
13	QWW	Female	22	Student
14	ZSP	Female	20	Barber
15	NLH	Female	23	Clerk
16	CFY	Female	30	Engineer
17	ZZH	Male	26	Clerk
18	LQY	Female	28	Guide
19	ZJL	Male	31	Teacher
20	YMY	Female	21	Student

In addition, a total of 965 questionnaires were collected in this study, and 45 unqualified questionnaires were removed (e.g., messy answers and omissions, less time spent, inconsistencies, etc.), and finally 920 valid questionnaires were obtained, with a qualified rate of 95.3%. Among them, there were 347 male samples, accounting for 37.72%; 573 female samples, accounting for 62.28%. The age of the samples was concentrated between 24 and 29 years old, of which 286 were aged 18–23 years old, accounting for 31.09%; 419 were aged 24–29 years old, accounting for 45.54%; and 215 were aged 30–35 years old, accounting for 23.37%. The basic demographic variables table is shown in Table 2.

**Table 2:** Statistical table of basic information of effective samples.

Statistical items	Specific content	Statistical value	Percentage
Gender	Male	347	37.72%
	Female	573	62.28%
Age	18–23	286	31.09%
	24–29	419	45.54%
	30–35	215	23.37%
educational background	High School and below	62	6.74%
	Undergraduate	559	60.76%
	Master and above	299	32.50%
Living Location	Urban	618	67.17%
	Rural	302	32.83%

## Data analysis

### Variable measurements and reliability testing

This study used IBM SPSS Statistics 25 for data analysis, and reliability analysis was performed by calculating the Cronbach Alpha coefficient of the scale by the software to determine the stability of the scale. Validity analysis was performed mainly based on KMO values and Bartlett’s sphere test. The reliability of the total scale was 0.847, and the reliability of the subscales were all higher than 0.7 and mostly concentrated above 0.8, indicating that the internal consistency of the scales in the questionnaire was high and the reliability of the scales was ideal. The KMO values of all variables were above 0.75 and the Bartlett sphere test sig values were all 0.000, indicating that the validity of the scales was high. Meanwhile, the data sources in this study were more diverse, so it was necessary to use Harman’s one-way analysis of variance for common method deviation test, and the results showed that the eigenvalues of 17 factors were >1, and the variance explained by the first common factor was 29.87%, which was less than the standard value of 40%, so there was no common method deviation, and the findings were valid for subsequent data analysis, and the scales (excerpts) The results of the reliability and validity analysis are shown in Table 3.

**Table 3:** Items and reliability and validity of the scale (Incomplete).

Variable	Code	Measuring project	Cronbach’s Alpha	KMO
Selfie Behavior	A1	I like to take selfies in my daily life	0.884	0.708
	A2	I will post my selfies on social media		
	A3	I spend a lot of time choosing the right angle for my selfies		
	A4	I will spend a lot of time on photo editing behavior		
	A5	I take selfies alone		
	A6	I will take selfies with friends and family		
	A7	I have a hard time choosing my selfies		
	A8	I take selfies by learning to imitate the style of good selfies on the Internet		
Social Comparison	B1	In my daily life, I like to compare myself with people who do things better than I do	0.823	0.747
	B2	When considering whether I can do something well, I compare myself to people who are better than me		
	B3	When you follow other people’s selfies in social media, you associate your appearance with your own		
	B4	When you follow other people’s selfies in social media, you associate your own appearance (including body, face, etc.) with the photos. Face, etc. with the person in the photo		
	B5	I often compare myself to others in terms of social (social skills, popularity)		
	B6	I do not compare my social media posts with those of others		
	B7	When things get bad, I often think of people who are doing better than me in social media.		
	B8	I always want to know what others would do in a similar situation		
	B9	I always pay close attention to the differences between the way I do things and the way others do things		
	B10	I never compare situations in my life to others		
Body Image	C1	I am always worried about being too fat or gaining weight	0.781	0.704
	C2	I often need to control my diet to lose weight		
	C3	My body is sexy and attractive		
	C4	I like the way I look now		
	C5	I can control my health		
	C6	I am happy enough with my appearance		
	C7	I do not care what people think of me on the outside		
	C8	I always try to improve my appearance		
	C9	I do not care about the state of my health in my life		
	C10	I do not care how my clothes look on my body		
Social Anxiety	D1	I am shy when I am in the middle of strangers	0.902	0.816
	D2	I feel that people will talk about me behind my back		
	D3	I get nervous when talking to people I do not know well		
	D4	I am reluctant to ask people to do things with me because I am afraid of rejection		
	D5	I feel nervous when I am with certain people		
	D6	I always feel like people are making fun of me		
	D7	I want to feel more confident in social situations		
	D8	I do not feel nervous and uncomfortable in social situations		

### Differences in demographic variables

In order to examine the differences in each variable in terms of gender, education level, and location of living, independent sample *t*-tests were conducted for social media selfie behavior, social comparison, body image, and social anxiety. On the variable of social media selfie behavior, there was a significant difference between the two groups of men and women (*T* = 1.72, *p* < 0.05), while the results of the mean comparison showed that the female youth group had a higher level of selfie behavior than men; on the variable of body image, there was a significant difference between the two groups of men and women (*T* = −0.64, *p* < 0.01), while the results of the mean comparison showed that men had a higher body image In the variable of social anxiety, there was a significant difference between the two groups (*T* = −0.52, *p* < 0.05), while from the results of the mean comparison, the level of social anxiety was higher in females than in males. There was no significant difference in the variables in terms of education level, but in the analysis of variability in terms of location of life, there was a significant difference in the variable of social comparison between urban and rural groups (*T* = −1.34, *p* < 0.01), while the results of the mean comparison showed that the level of social comparison was higher in the urban youth group than in the rural youth group; in the variable of social anxiety, there was a significant difference between urban and rural groups In the variable of social anxiety, there was a significant difference between the urban and rural groups (*T* = 0.75, *p* < 0.01), while from the results of mean comparison, the degree of social anxiety was higher in the urban youth group than in the rural youth group. The specific results of the analysis are shown in Table 4.

**Table 4:** Analysis of differences among variables in gender, education level, and living location.

Test for differences in each variable on gender							
Variables	Male (*n* = 347)		Female (*n* = 573)		*t*		
	*M*	SD	*M*	SD			
Social media selfie behavior	3.02	0.87	5.41	0.42	1.72^*^		
2. Social comparison	4.69	0.71	5.06	0.43	1.55		
3. Body image	3.12	0.82	2.66	0.31	−0.64^**^		
4. Social anxiety	4.17	0.58	4.76	0.67	−0.52^*^		
**Test for differences in educational attainment for each variable**							
**Variables**	**High School and below (*n* = 62)**		**Undergraduate (*n* = 559)**		**Master and above (*n* = 299)**		***t***
	***M***	**SD**	***M***	**SD**	***M***	**SD**	
Social media selfie behavior	4.02	1.07	4.41	1.18	4.01	1.31	0.62
2. Social comparison	3.79	0.84	4.36	0.86	5.01	0.92	1.28
3. Body imagery	3.05	0.71	2.78	0.31	2.14	0.90	1.45
4. Social anxiety	4.49	0.56	4.78	0.61	5.17	0.64	1.02
**Test for differences in variables on living location**							
**Variables**	**Urban (*n* = 618)**		**Rural (*n* = 302)**		***t***		
	***M***	**SD**	***M***	**SD**			
Social media selfie behavior	4.64	0.64	4.09	0.62	0.92		
2. Social comparison	5.02	0.57	4.15	0.53	−1.34^**^		
3. Body imagery	2.15	0.69	2.76	0.63	0.52		
4. Social anxiety	4.59	0.75	4.03	0.70	0.75^**^		

^*^p < 0.05; ^**^p < 0.01.

### Correlation analysis

The Pearson correlation coefficient is a measure of the linear relationship between a fixed-range variable and the dependent variable, which is also a fixed-range variable. The correlation coefficient ranges between −1 and +1, with positive values representing positive correlations and negative values representing negative correlations. The correlation analysis revealed significant correlations between the variables. According to the data in Table 5, social media selfie behavior (*M* = 4.185, SD = 0.835), social comparison (*M* = 4.697, SD = 3.772), body image (*M* = 2.420, SD = 0.829), and social anxiety (*M* = 5.115, SD = 1.475). The specific results of the analysis are shown in Table 5.

**Table 5:** Table of descriptive statistics results and variable correlations.

Variables	*M*	SD	1	2	3	4
1. Social media selfie behavior	4.185	0.835	1			
2. Social comparison	4.697	3.772	0.073^**^	1		
3. Body image	2.420	0.829	−0.152^**^	−0.213^*^	1	
4. Social anxiety	5.115	1.475	0.136^**^	0.537^**^	−0.049^*^	1

^*^p < 0.05; ^**^p < 0.01.

### An examination of the mediating role of social comparison and body image

This study examined the mediating effect of social comparison and body image in the relationship between social media selfie behaviors and social anxiety using the bias-corrected percentile Bootstrap method. The effect sizes and 95% CIs of the mediating effects of social comparison and body image on social media selfie-related behaviors and social anxiety were estimated using the SPSS macro program developed by Hayes by taking 5,000 samples to estimate the 95% confidence intervals (95%CIs) of the mediating effects. Regression analysis showed that social media selfie behavior was a direct positive predictor of social anxiety (*β* = 0.429, *p* < 0.01); social media selfie behavior was a direct positive predictor of social comparison (*β* = 0.190, *p* < 0.01); social media selfie behavior and social comparison were negative predictors of body image (*β* = −0.082, *p* < 0.05; *β* = −0.068, *p* < 0.01); after all variables were included in the regression equation, social media selfie behavior directly positively predicted social anxiety (*β* = 0.251, *p* < 0.05), social comparison positively predicted social anxiety (*β* = 0.537, *p* < 0.01), and body image negatively predicted social anxiety (*β* = −0.026, *p* < 0.01).The specific results of the analysis are shown in Table 6.

**Table 6:** Table of regression analysis of the relationship between variables.

Regression equation		Overall fit index			Significance of regression coefficients		
Result variables	Predictive variables	*R*	*R* ^2^	*F*	*β*	*t*	*p*
Social anxiety	Social media selfie behavior	0.029	0.040	12.035^**^	0.429	2.372^**^	0.004
Social comparison	Social media selfie behavior	0.305	0.261	9.506^*^	0.190	2.525^**^	0.007
Body image	Social media selfie behavior	0.226	0.035	7.264^**^	−0.082	−1.286^*^	0.03
Social anxiety	Social comparison				−0.068	−2.059^**^	0.002
	Social media selfie behavior	0.512	0.314	9.243^**^	0.251	3.259^*^	0.04
	Social comparison				0.537	4.407^**^	0.005
	Body image				−0.026	−1.319^**^	0.004

^*^p < 0.05; ^**^p < 0.01.

The results of the bias-corrected percentile Bootstrap method mediated effect analysis showed that social comparison and body image mediated the relationship between social media selfie behavior and social anxiety, with a mediated effect value of 0.604, accounting for 42.29% of the total effect of social media selfie behavior on social anxiety. Specifically, the total mediating effect consisted of indirect effects of three pathways: indirect effect 1 (0.196) through the pathway of social media selfie behavior → social comparison → social anxiety, Bootstrap 95% CI did not contain 0, indicating that the mediating effect of social comparison in the relationship between social media selfie behavior and social anxiety was significant; The indirect effect 2 (0.073) through the social media selfie behavior → social comparison → body image → social anxiety pathway, Bootstrap 95% CI does not contain 0, indicating a significant mediating role of social comparison and body image in the chain between social media selfie behavior and social anxiety relationship; the indirect effect 3 (0.335) through the social media selfie behavior → body image → social anxiety pathway, Bootstrap The 95% CI did not contain 0, indicating that the mediating role of body image in the relationship between social media selfie behavior and social anxiety was significant. The three indirect effects accounted for 12.08%, 7.76%, and 22.45% of the total effects, respectively, and the results of the analysis are shown in Table 7.

**Table 7:** Table of results of sequential mediated effects analysis.

	Indirect effect value	Bootstrap SE	Bootstrapping 95% CI		Relative indirect effects (%)
			Lower-bound	Upper-bound	
Total mediating effect	0.604	0.075	0.117	0.751	42.29
Indirect effect 1	0.196	0.037	0.071	0.147	12.08
Indirect effect 2	0.073	0.092	0.006	0.518	7.76
Indirect effect 3	0.335	0.048	0.035	0.077	22.45

**Figure 1:**
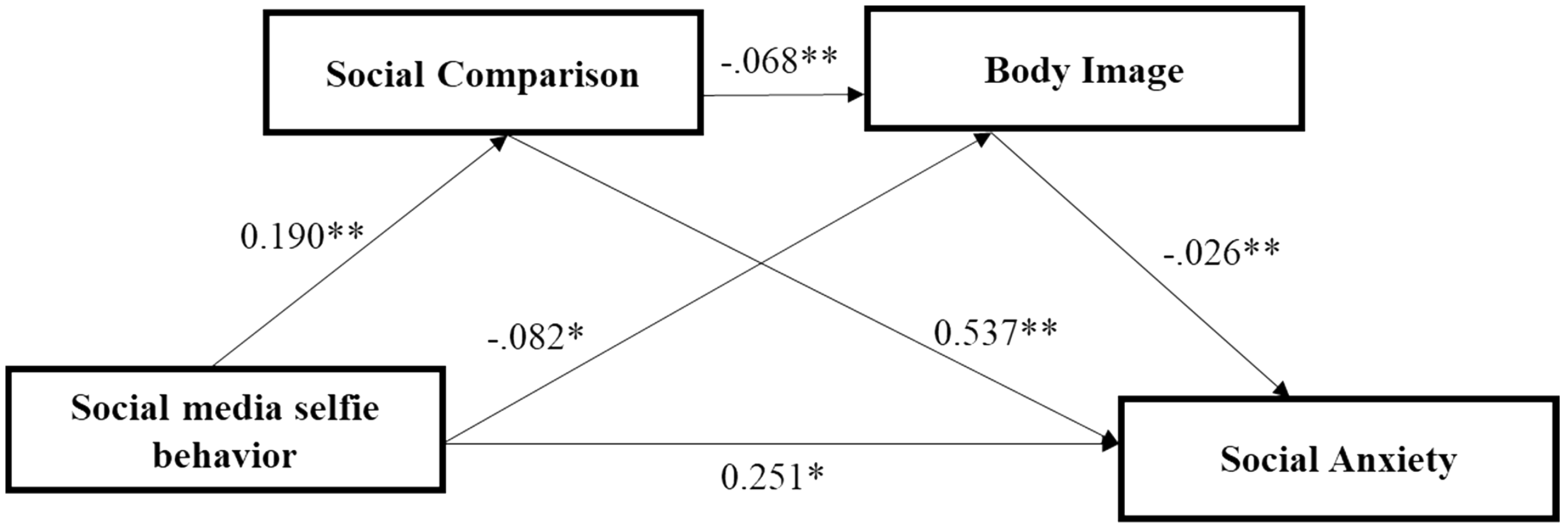
Process-conditional model of the association between social media selfie behavior and social anxiety. *p < 0.05; **p < 0.01.

Based on the analysis of the empirical data, hypotheses H1, H2, H3, H4, H5, H6, and H7 are supported and the adjusted model path coefficients are plotted as shown in Figure 1.

## Research conclusion

### Differentiated discussion of demographic variables

This study found that the female youth group had a higher degree of selfie behavior than males, males had a higher degree of body image evaluation than females, and females had a higher degree of social anxiety than males. Meanwhile, the urban youth group had higher levels of social comparison and social anxiety than the rural youth group. The contemporary empirical studies on selfie behavior focus on the female group because of the positive self-presentation behavior that accompanies women in social media, which also validates the previous studies on selfie in the female group, and the underlying reason is that they are more emotional and like to show and record themselves, and compared with boys, girls post more personal status on social networks and are more inclined to post information related to body image, such as selfies etc. They are more concerned about the image they present and how they are perceived by others, which is one of the manifestations of women’s self-objectification. They are more prone to social comparison, and existing research demonstrates that repeated exposure to media sexual objectification causes women to habitually view themselves through the lens of a third party, frequently self-monitor their appearance, and be more sensitive to body-related information (Fredrickson and Roberts, 1997). At the same time, research has demonstrated that men have higher body image ratings than women, suggesting that there are gender differences in body image ratings, and that overall women show more weight concerns and body dissatisfaction than men, which may also validate previous findings of differences in body image ratings between men and women (Paxton et al., 2006). In addition, in the comparison between urban and rural youth, urban youth showed higher levels of social comparison and social anxiety than rural youth, analyzing the specific reasons, which may be related to the social development and urban–rural environmental development differences in China. The sample of this study was a population of youth aged 18–35, with a large age span, and different age groups were exposed to media technology either earlier or later, and urban youth and younger individuals would preferentially exposed to new things. In addition, there is a gap between the growing environment of urban youth and rural youth, as rural areas are relatively slower in the development of new things, and the population movement and new things change less than urban areas. Urban youths are relatively open-minded and have a higher pursuit of material conditions, and they have a higher social comparison mentality. Meanwhile, facing the huge pressure of survival in the city, social media and other media technologies become a haven for them to escape from reality and relax. In contrast, rural youths live in a rural society and must interact closely with their relatives, friends and neighbors in their daily lives, which may be an important reason why their social anxiety level is lower than that of urban youths.

### Selfies trigger anxiety? The relationship between selfie behavior and social anxiety

The empirical findings prove that the degree of selfie behavior in youth group positively predicts social anxiety. Through the participant observation and interviews we found that image socialization has been integrated into our daily life, and selfies are considered an important form of online self-presentation and impression management. People upload selfies for three main purposes: to complete self-appreciation; to show the best self-image to others; and to get more likes, comments and attention. Therefore, people will often choose to take or post the most perfect image. The interviews revealed that usually a good selfie requires complex processing, and for those who like to take selfies, it is crucial to select and edit the selfie, and when the painstaking selfie posted to social media does not get the desired likes and comments, it means that the selfie taker does not get the desired attention and affirmation, and they may try to present the perfect self. They may retouch their selfies according to the socially acceptable “ideal beauty” in order to present a “perfect” version of themselves, thereby attracting the attention of others and gaining psychological satisfaction. In contrast, if the selfie taker receives praise and affirmation of his or her appearance, he or she may reinforce the selfie-taking behavior. In this regard, selfies are motivated in large part by the desire to put one’s best self on display and obtain the best evaluation from others. But drastic selfies or retouching may lead to the viewer’s perception that the publisher is acting insincerely online, thus failing to generate widespread approval and satisfaction of the selfie from others. This runs counter to the purpose of going to the trouble of retouching the picture in the first place, and it is clear that such selfies cannot serve the purpose of good identity impression management in social media, and individuals may experience reduced body satisfaction and anxiety due to the inconsistency of their online and offline images, which in turn can lead to adverse emotions such as social fatigue and social anxiety (Brown and Tiggemann, 2016). This finding answers the question posed in Research Question one (Q1).

### Sequential mediation of social comparison and body image

The empirical data demonstrated that social comparison and body image have a sequential mediating role in the path of social media selfie behavior on social anxiety. This finding uncovered deep-seated psychological mechanisms of individuals in the process from social media behavior to social emotions. In the youth group’s selfie behavior, “physical appearance” is the focus of their attention, and therefore their social anxiety is directed to their body image. This may cause youth to focus more on their lack of appearance when taking selfies and experience more appearance anxiety, which may lead to negative body image and social anxiety (Holland and Tiggemann, 2016). In addition, people tend to choose idealized parts of themselves and embellish their images to some extent as they engage in self-disclosure on social media. When individuals engage in behavioral activities related to social media platforms, such as taking selfies, they unconsciously generate social comparisons, and the more likely they are to look at themselves with the harsh set of esthetic standards promoted on social media, the more likely they are to develop negative emotions such as dissatisfaction with their appearance (Tiggemann and Barbato, 2018).

At the same time, this study included measures of body information among the measures of social comparison, and such comparisons can lead individuals to hold negative evaluations of their body image, and then they may worry about their image and performance in social interactions, and thus experience social anxiety. This study confirmed the significant mechanism between social comparison and body image, which resulted in the emergence of social media selfies as a predictor of social comparison, and social comparison as an influence on body image, which in turn increases social anxiety in individuals. This finding answers the question posed in research question two (Q2). Taken together, the multiple mediating effects of social comparison and body image found in this study are empirically and theoretically meaningful.

## Discussion

In this study, we found that young people have more negative body image and higher social anxiety. In fact, along with the rapid development of modern Chinese society, young people have left their hometowns for a long time and set out for the big cities to strive for greater personal development opportunities and growth space. Through observations and interviews, we found that most young people have a limited range of interpersonal activities, an inner emptiness, a lack of emotional comfort and companionship, a heavy pressure of life and work, and a negative social mentality with a strong sense of anxiety and loneliness. Facing the impact of the Corona Virus Disease on the work, study, socialization and life of the youth group, and the sudden isolation will make them more anxious and depressed (Brooks et al., 2020). However, the development of media technology provides a convenient channel for young people to escape from real life, and they are willing to immerse themselves in the Internet to relieve the anxiety and anxiety of real social interactions, and the Internet has become an important field for modern young people’s emotional support and catharsis (Bennett et al., 2008). At the same time, in the process of seeking breakthroughs, young people are eager to seek social identity from new spaces such as the Internet. They freely transmit information in the online world, are willing to share and show themselves, and are empowered by social media to have the opportunity to show their bodies and construct gender temperament in the form of words, pictures and videos, actively participate in personal and other people’s body practices and exchange their body experiences with each other. This inevitably has a tremendous impact on individuals’ body constructions and psycho-emotions. Young people rely heavily on canonic binaries from utopian and dystopian interpretations of networked technologies to apply labels to themselves, they are deeply influenced by the online media, which can also reflect the dialectical struggle they are always experiencing in their daily lives, triggering their ambivalence and anxiety (Tiidenberg et al., 2017). Technological advances have provided more freedom of choice, but the flood of information has also thrown individuals into a state of uncertainty and anxiety, leading to a highly sensitive state of mind and a general group anxiety among contemporary youth (Fox and Moreland, 2015). In the present study, the causal relationship between social comparison and social anxiety more directly links group pressure to youth anxiety.

By examining the social media selfies, social comparison, body image and social anxiety of the youth group, this study demonstrates that the development of media technology has greatly influenced people’s social interactions, and that social media selfies provide the means of expression and technological tools for the fictionalization and exhibition of the youth group’s role identity. Through observation, we found that one of the important motives of individual selfie behavior is life logging, and at the same time, this kind of selfie image that expresses oneself in a given way focuses on performance or ideal self-expression, which enables the youth group to realize the mental world or emotional world in the virtual network space under the pressure of realistic anxiety and life pressure to achieve self-revelation, and present the self-expression with the help of media technology. Everyone has the right to record their life and show themselves, and the act of selfie image publishing and photo editing reflects the individual’s creative life more, making information dissemination more diversified and autonomous (Weilenmann and Hillman, 2020). Individuals’ image shaping in social media is graspable, and selfie photos become the key to individuals’ self-shaping as well as forming good impression management, reflecting a higher degree of motivation in online interactions. In the online environment, both parties in the interaction have different degrees of role expectations for themselves and others, and when the real self and the ideal self-have large differences, the self-identity will fall into a state of dissonance, and although this dynamism gives individuals more choices, it directly affects the formation of the impetuous mentality of interaction thus leading individuals into anxiety (Boyraz and Kuhl, 2015). This can also explain the empirical findings of this study that social media selfie behavior triggers social anxiety to some extent.

This study found that social comparison and body image mediated significantly in the model pathway, and a high degree of social comparison mentality and negative body image was prevalent among the youth group. The interviewees indicated that when individuals posted selfies of their own image and status in the social media field, they formed an invisible interaction of “seeing” and “being seen” in the virtual social media, and by paying attention to the likes and comments of others on their own postings, they formed an objectified social comparison mentality. By following the interaction of others’ likes and comments on one’s own postings, an objectified social comparison psychology is formed, and individuals become dissatisfied with their own bodies by comparing themselves with acquaintances or strangers who have better physical appearance than themselves (Moya-Garofano and Moya, 2019). Meanwhile, the ongoing epidemic has led to a dramatic shift in the way people communicate. In order to maintain daily contact with others, the public is increasingly using online meetings such as social media to keep in touch, and research has found similarities between online video calls and taking and posting “selfies” on social media. Because during video calls, individuals are required to look at their appearance for long periods of time as if they were looking in a mirror, the results of using video calls are the same as presenting selfies on social media, with negative effects on body image (Pikoos et al., 2021). In addition, the empirical findings of this paper show that image information in social media has become an indispensable component in communicating interpersonal relationships, and to a certain extent, it can also prove that individuals in digital existence will generate new social relationship needs, and with the support of media technology, non-verbal communication through image symbols has become a new trend. Through observations and interviews we found that the youth group spends too much time on social media, and the high degree of selfie behavior increases the individual’s focus on self, triggers a higher social comparison mentality and negative body image, which leads to the emotion of social anxiety, so they reduce the offline activities of face-to-face socializing, and such social media behavior is harmful to people’s emotional, psychological, and interpersonal relationships (Pawijit et al., 2019).

## Strengths

There are still few studies on the potential influence mechanisms of selfie behaviors on social anxiety in youth group, and almost no articles in China have conducted systematic research demonstrating the influence and mechanism of action of selfie behaviors on social anxiety. It is of great significance to maintain social stability and harmony to understand the changes brought by technological innovation and institutional transformation to the society and even to the group itself, and then to treat these changes correctly and avoid the risks. This study explores the relationship between selfie behaviors and social psychology of youth group, and the existence of multiple mediating mechanisms, and explores the basic research framework of selfie behaviors-social comparison-body image-social anxiety from both theoretical and empirical perspectives. The research hypothesis and research model were empirically tested to further clarify the relationship between selfie behavior and social psychological motivation, and to lay the theoretical and empirical foundation for subsequent research.

## Limitations

The empirical findings in this study have some value, but the scale design process only considered individuals as selfie information disseminators, while ignoring the perspective of information reception, the scope of the study group focused on the Chinese youth population, and there may be significant differences in selfie behavior and related social anxiety psychology among different populations, and also whether the empirical results can be adapted to the cultures of other countries needs further investigation and verification. In the future, the discussion on selfie behaviors needs to consider the identity of individuals as information receivers, increase the sample size of different age groups, and analyze the differences in how different groups face this issue, as well as further cross-cultural related studies to explore the similarities and differences in selfie posting behaviors and related consequences in different cultural contexts. In addition, the psychological motivation and behavior of selfies should be further refined in subsequent studies, and a detailed scale design and examination of social media selfie behaviors should be conducted. It is also necessary to consider the influence of media information technology and different publishing scenarios on individual self-construction. The empirical research related to selfies is still in its initial stage, and in future studies the causal relationship between selfie behavior and social psychology in the digital media era can be revealed more comprehensively, and more variables related to individual differences and psychological traits of youth group can be incorporated to explore the influence of selfie behavior on social psychology. In addition, experimental methods and more detailed and in-depth questionnaire items were attempted to make precise causal inferences, which need to be further explored and refined in future studies.

## Data availability statement

The original contributions presented in the study are included in the article/supplementary material, further inquiries can be directed to the corresponding author.

## Author contributions

YL, JZ, and JH contributed to the conception and design of the study. YL was responsible for the study design and framework, performed the statistical analysis, and wrote the first draft of the manuscript. JZ organized the database. JZ and JH wrote parts of the manuscript. All authors contributed to the article and approved the submitted version.

## Funding

This study is a phased achievement of Guangdong Province philosophy and social science planning project Research on prevention strategies of youth’s addiction to online social games (project number: GD20CMK06).

## Conflict of interest

The authors declare that the research was conducted in the absence of any commercial or financial relationships that could be construed as a potential conflict of interest.

## Publisher’s note

All claims expressed in this article are solely those of the authors and do not necessarily represent those of their affiliated organizations, or those of the publisher, the editors and the reviewers. Any product that may be evaluated in this article, or claim that may be made by its manufacturer, is not guaranteed or endorsed by the publisher.
